# Combined effects of warming and drought on plant biomass depend on plant woodiness and community type: a meta-analysis

**DOI:** 10.1098/rspb.2022.1178

**Published:** 2022-10-12

**Authors:** Rutger A. Wilschut, Jonathan R. De Long, Stefan Geisen, S. Emilia Hannula, Casper W. Quist, Basten Snoek, Katja Steinauer, E. R. Jasper Wubs, Qiang Yang, Madhav P. Thakur

**Affiliations:** ^1^ Ecology, Department of Biology, University of Konstanz, Universitätsstraße 10, Konstanz 78464, Germany; ^2^ Department of Terrestrial Ecology, Netherlands Institute of Ecology (NIOO-KNAW), Wageningen 6708 PB, The Netherlands; ^3^ Louis Bolk Institute, Kosterijland 3-5, Bunnik 3981 AJ, The Netherlands; ^4^ Laboratory of Nematology, Wageningen University, Wageningen 6708 PB, The Netherlands; ^5^ Biosystematics Group, Wageningen University, Wageningen 6708 PB, The Netherlands; ^6^ Institute of Environmental Sciences, Leiden University, Einsteinweg 2, Leiden 2333CC, The Netherlands; ^7^ Theoretical Biology and Bioinformatics, Utrecht University, Padualaan 8, Utrecht 3584 CH, The Netherlands; ^8^ Sustainable Agroecosystems Group, Department of Environmental Systems Science, ETH Zürich, Universitätstrasse 2, Zürich 8092, Switzerland; ^9^ State Key Laboratory of Grassland Agro-Ecosystems, College of Ecology, Lanzhou University, Lanzhou, Gansu 730000, People's Republic of China; ^10^ Institute of Ecology and Evolution and Oeschger Centre for Climate Change Research, University of Bern, Bern 3012, Switzerland

**Keywords:** climate warming, precipitation increase, precipitation decrease, global change experiments, aboveground plant biomass, belowground plant biomass

## Abstract

Global warming and precipitation extremes (drought or increased precipitation) strongly affect plant primary production and thereby terrestrial ecosystem functioning. Recent syntheses show that combined effects of warming and precipitation extremes on plant biomass are generally additive, while individual experiments often show interactive effects, indicating that combined effects are more negative or positive than expected based on the effects of single factors. Here, we examined whether variation in biomass responses to single and combined effects of warming and precipitation extremes can be explained by plant growth form and community type. We performed a meta-analysis of 37 studies, which experimentally crossed warming and precipitation treatments, to test whether biomass responses to combined effects of warming and precipitation extremes depended on plant woodiness and community type (monocultures versus mixtures). Our results confirmed that the effects of warming and precipitation extremes were overall additive. However, combined effects of warming and drought on above- and belowground biomass were less negative in woody- than in herbaceous plant systems and more negative in plant mixtures than in monocultures. We further show that drought effects on plant biomass were more negative in greenhouse- than in field studies, suggesting that greenhouse experiments may overstate drought effects in the field. Our results highlight the importance of plant system characteristics to better understand plant responses to climate change.

## 1. Introduction

Anthropogenic climate change is affecting terrestrial ecosystems worldwide [1–3]. As primary producers, plants form the base of both above- and belowground food webs [4,5] and as such play a central role in carbon cycling and overall ecosystem function. Our ability to predict ecosystem responses to climate change therefore strongly depends on how above- and belowground plant biomass are affected [6,7]. Recent syntheses have indicated that climate warming and precipitation extremes (precipitation decreases, hereafter called ‘droughts’, or precipitation increases) are among the most pressing forms of anthropogenic climate change influencing the performance of terrestrial plants [8–10]. Moreover, different climate change factors can interactively affect plant performance, by either amplifying or dampening each other's positive or negative effect on plant biomass (also called multiplicative or nonlinear effects; [8,11,12]). However, in contrast with single effects of warming and precipitation extremes, relatively little is known about how combined effects of warming and precipitation extremes affect plant performance above- and belowground, and in particular, which factors may underlie the magnitude of such combined effects.

In terrestrial ecosystems, precipitation extremes are typically known to affect plant performance [9]. Recent meta-analyses indicated that summer droughts decrease terrestrial plant performance across habitats and that impacts of drought are stronger than the impacts of other predicted global change effects [10,13]. By contrast, precipitation increases, as well as warming, typically enhance plant performance [8,10,13]. While multi-factorial global change experiments have shown that warming and precipitation extremes can interactively affect plant performance [14,15], several meta-analyses detected additive combined effects (warming + precipitation extremes) rather than interactive combined effects (warming × precipitation extremes) [8,10,13]. However, these meta-analyses quantified overall interaction effect sizes based on datasets in which studies that separately examined the effects of warming or precipitation extremes were pooled with studies that examined both single and combined effects of warming and precipitation extremes [10,13]. Such pooling of studies with different experimental designs possibly could have obscured the detection of interactive effects. Moreover, to date, meta-analyses have mainly used abiotic variables (e.g. climatic conditions) to explain variation in (combined) effects of warming and precipitation extremes on terrestrial plant performance [8,10,13]. However, in addition to such abiotic effects, biotic characteristics of the study system may further help to explain the variation of single and combined effect sizes of warming and precipitation extremes on plant performance.

Plant responses to drought and warming may depend on adaptive strategies to overcome water shortages and thermal stress that are related to plant woodiness [16,17]. For instance, due to failures of the hydraulic system, droughts can have long-lasting negative impacts on woody plants and reduce their survival, but such impacts may not be immediately reflected in their biomass responses to drought [3,18]. By contrast, herbaceous plants often show immediate biomass responses to drought but may also recover quickly after the drought period is over [19,20]. These responses to drought may vary between plant shoots and roots given the differences in resource economics between these compartments, as well as among plant species, for example owing to variation in symbiotic relationships with soil microorganisms that help to maintain water uptake under dry conditions [21–23]. Moreover, root responses to warming for both woody plants and herbaceous plants could differ from shoot responses given that temperature buffering in soils is often higher than in air [24–26]. However, it is likely that warming effects on plant roots may be magnified in soils with limited water availability [27], as drought has consistently been shown to alter root growth and root resource uptake from the soil [28–31]. In this respect, it is also important to consider that plant responses to drought may also differ between field and greenhouse studies. For instance, plants in field conditions may be better able to show plastic responses, such as an increase in rooting depth, whereas plants grown in pots in greenhouse studies may be limited in their ability to exhibit trait plasticity to overcome abiotic stresses [32]. Moreover, compared to plants growing in the field, plants growing in pots may be affected by the limited capacity of their respective soils to buffer abiotic changes (e.g. increased temperatures) [33].

Plant monocultures and diverse plant communities are also likely to differ in their responses to warming and precipitation extremes, given that plant diversity often mediates negative environmental impacts and generally enhances plant community productivity [34,35]. Warming, for example, increased aboveground biomass in diverse plant communities compared to monocultures, although such an effect was not found for belowground biomass [36]. Moreover, communities with a high diversity of plant species typically are better able to maintain community productivity during drought events than low-diversity communities [37–39]. Such differences in drought resistance between low- and high-diversity communities may occur owing to stronger drought-ameliorating effects of belowground mutualists in diverse plant communities [40], as well as owing to an increasing probability of the presence of species with effective responses against drought in diverse communities compared to monocultures [41,42].

Here, we examine how combined experimental treatments of warming and drought or warming and increased precipitation affect terrestrial plant biomass both above- and belowground. We performed a meta-analysis using above- and belowground plant biomass data from studies in which warming- and precipitation changes were experimentally crossed, and associated the variation in single and combined effect sizes with two potential key biotic variables: plant woodiness and plant community type. We specifically test two hypotheses: (i) overall, combined effects of warming and precipitation extremes on above- and belowground plant biomass are additive (i.e. lack of amplifying and/or dampening interactive effects), although we expect that the effect sizes could be stronger for aboveground biomass than for belowground biomass and (ii) plant woodiness and plant community type (monoculture versus mixed) explain variation in both single and combined effects of warming and precipitation extremes on above- and belowground biomass. Finally, to understand whether variation in plant responses to warming and precipitation extremes in part may be determined by methodological approaches, we tested the hypothesis that (iii) single and combined effect size variability depend on whether a study was performed in the greenhouse or in the field.

## 2. Material and methods

### (a) Literature search and data collection

We performed a meta-analysis with data from two types of full-factorial experimental studies, which either examined above- and/or belowground plant biomass responses to experimental warming and drought or to experimental warming and increased precipitation. We report the meta-analyses of these two kinds of studies separately given the differences in their experimental design. We conducted a literature search in ISI Web of Science Core Collection on 11 January 2021 (cut-off date) using the following terms: (‘Temperature’ OR ‘Warming’ OR ‘Heat’) AND (‘Drought’ OR ‘Precipitation’ OR ‘Rainfall’ OR ‘Moisture’ OR ‘Flood*’) AND (‘Plant biomass’ OR ‘Plant product*’ OR ‘Shoot biomass’ OR ‘Root biomass’ OR ‘Aboveground biomass’ OR ‘Belowground biomass’ OR ‘Plant cover’) AND (‘Experiment*’ OR ‘Manipulat*’). The search was carried out for ‘All Fields’ in ISI Web of Science, which resulted in a total of 682 studies (with their title, abstract, author keywords and Keywords Plus) that were subsequently screened for experimental studies from which we could extract data. We first screened papers for the presence of experimental data on interactive effects of warming and precipitation extremes (drought or precipitation increase or both). Papers that met these criteria were then screened for the presence of above- and/or belowground plant biomass data. We were eventually able to extract biomass data from 37 independent, full-factorial experimental studies that measured aboveground and/or belowground plant biomass (also see PRISMA diagram in electronic supplementary material, figure S1). There were 27 studies experimentally applying warming and drought treatments and 19 studies experimentally applying warming and increased precipitation treatments in our meta-analysis. Some studies applied both drought and increased precipitation in their experiments (electronic supplementary material, data S1). Temperature increases in studies combining warming and drought ranged from +0.5°C to +10°C (average +4°C) and from +0.68°C to +11.4°C (average +3°C) in studies combining warming and increased precipitation. Experimental drought treatments (precipitation decreases) ranged from −92% to −5.54% (average −35.2%), while precipitation increases ranged from +15% to +203.4% (average +84.4%).

The data from each study were extracted from figures, tables or main text. We extracted mean, s.d. and sample sizes for above- and/or belowground plant biomass from all combinations of warming and precipitation extreme treatments in each study (electronic supplementary material, data S1). To extract the data from the figures, we either used Plot Digitizer [43] or ImageJ [44]. We obtained aboveground biomass (or shoot biomass) from 29 studies and belowground biomass from 17 studies across warming*drought and warming*increased precipitation interaction studies (electronic supplementary material, data S1).

### (b) Data analysis

We took a two-step approach in our meta-analysis. First, we estimated single effects of warming and precipitation extremes, and their interaction effects, on plant biomass. We performed this separately for above- and belowground plant biomass. Second, we used a set of moderator variables to examine what may explain the variation in effect sizes obtained in step 1.

We estimated the individual effects of warming and precipitation extremes on aboveground and belowground plant biomass using the Hedge's *g* effect size [45], with corrections for small sample sizes. The Hedge's *g* were calculated using the following formula:

$$Hedge{\;}^{\prime }\;s\;g(individual)=\frac{{\mathrm{Biomass}}_{T}\;-{\mathrm{Biomass}}_{C}\;}{S}\times J,$$

where Biomass*_T_* is aboveground or belowground (mean) biomass in warming or precipitation regime treatment(s) and Biomass*_C_* is aboveground or belowground (mean) biomass in respective control treatments. *S* is the pooled s.d. and is estimated as follows:

$$S=\sqrt{\frac{(N_{T}-1)\;\times \;\sigma_{T\;}^{2}+(N_{C}-1)\;\times \;\sigma_{C\;}^{2}}{N_{T}+N_{C}-1}},$$

where *N_T_* stands for the sample size of the given treatment and *N_C_* is the sample size of the respective control. $\sigma_{T\;}^{2}$ and $\sigma_{C\;}^{2}$ are variance of treatment and controls, respectively. *J* is the correction factor for small sample sizes and is calculated as

$$J=1-\;\frac{3}{4\;\times (\;N_{T}+N_{C})-1}.$$

To estimate the interaction effect sizes, we also used Hedge's *g* with the following formula used in several previous studies [10,13,46,47]:

$$\begin{array}{rl} & Hedge^{\prime }s\;g(combined)\\ & \;=\frac{({\mathrm{Biomass}}_{AB}\;-{\mathrm{Biomass}}_{A})\;\times ({\mathrm{Biomass}}_{B}\;-{\mathrm{Biomass}}_{C})\;}{2\times S^{i}}\times J^{i}, \end{array}$$

where Biomass*_AB_* is the (mean) aboveground or belowground biomass in the treatment combination of warming and drought or the treatment combination of warming and increased precipitation. Biomass*_A_* and Biomass*_B_* are (mean) aboveground or belowground biomass in warming and precipitation extreme treatments, respectively. Biomass*_C_* stands for (mean) aboveground or belowground biomass in control treatments. *S^i^* and *J^i^* are the pooled standard deviations and correction term for sample bias, respectively, which were estimated as following.

$$S^{i}=\sqrt{\frac{(N_{C}-1)\;\times \;\sigma_{C\;}^{2}+(N_{A}-1)\;\times \;\sigma_{A}^{2}+(N_{B}-1)\;\times \;\sigma_{B\;}^{2}+(N_{AB}-1)\;\times \;\sigma_{AB}^{2}}{N_{C}+N_{A}+N_{B}+N_{AB}-4}}$$

and

$$J^{i}=1-\;\frac{3}{4\;\times (\;N_{C}+N_{A}+N_{B}+N_{AB})-1},$$

where *N_C_, N_A_, N_B_* and *N_AB_* are sample sizes, and $\sigma_{C\;}^{2}$, $\sigma_{A\;}^{2}$, $\sigma_{B\;}^{2}$ and $\sigma_{AB\;}^{2}$ are variance of control, warming, drought/increased precipitation, and combined treatments of warming and drought or warming and increased precipitation treatments, respectively. The variance of Hedge's *g* (interactive) was estimated using the following formula:

$$V=\left(\frac{1}{N_{C}}+\frac{1}{N_{A}}+\frac{1}{N_{B}}+\frac{1}{N_{AB}}+\;\frac{d^{2}}{2\times (\;N_{C}+N_{A}+N_{B}+N_{AB})}\right)\times \;\frac{1}{4},$$

where *d^2^* is the square of the weighted mean calculated as explained in Yue *et al.* [10].

We used random effects models for our meta-analysis, which allow flexibility in the variation of true effects from one study to the other [45]. Moreover, we always used study identity as a random intercept in all our meta-regression models to account for repeatability of single studies with multiple effect sizes. We used restricted maximum-likelihood estimators to obtain the effect size estimates and their variance owing to their efficiency in obtaining unbiased estimates [48]. The effect sizes were considered statistically significant when their 95% confidence intervals (CIs) did not overlap with zero. When the 95% CIs of interactive effect sizes overlapped with zero, combined effects were considered to be additive [47]. When not overlapping with zero, positive or negative interaction effects were considered to affect plant biomass more positively or negatively than expected based on the effects of single factors.

The total heterogeneity and its test statistics for each random effects model were further estimated to examine how heterogeneous Hedge's *g*'s were for aboveground or belowground biomass across studies [49]. A significant total heterogeneity (*p*-value < 0.05) indicates a greater among-study variance than expected when accounting for the sampling error within the random effects models. If this is the case, it indicates that additional unexamined factors influenced effect sizes and its variance. We also tested for the effects of publication bias in the estimation of effect sizes using contour funnel plots (electronic supplementary material, figure S2). Visual inspection of funnel plots showed minimal publication bias in our meta-analysis for both studies that examined warming and drought effects and studies that examined warming and increased precipitation effects (electronic supplementary material, figure S2).

In our second step, we used moderator analysis to test the effects of four variables (‘aboveground versus belowground’, ‘woody versus herbaceous’, ‘monoculture versus mixture’ and ‘field versus greenhouse’) on both additive and interactive effects of warming and precipitation extremes on plant biomass. The importance of each moderator for a given effect size from the experiments combining warming and drought or experiments combining warming and increased precipitation was estimated from the sum of Akaike weights [50]. All our analyses were carried out in R statistical software v. 4.1.0 [51], using the metafor package [52] for random effects models and multi-level meta-analysis. The sum of Akaike weights was estimated using the MuMin package [53].

## 3. Results

**Figure 1.**
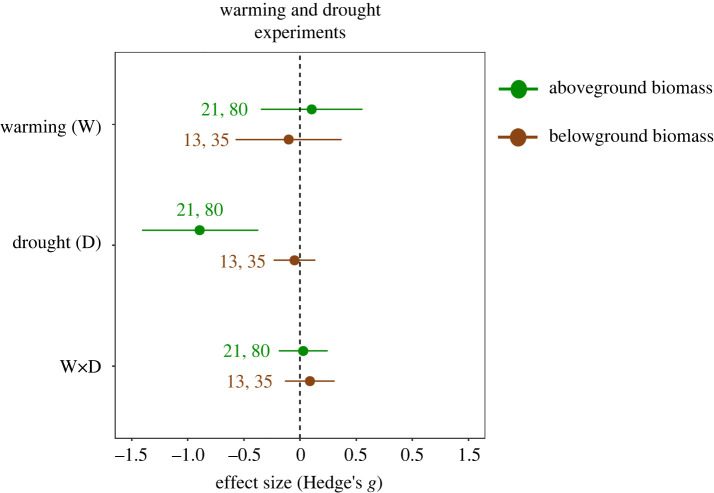
Mean effect sizes ± 95% CIs for experimental warming and drought effects on aboveground (upper effect sizes) and belowground (lower effect sizes) plant biomass. Warming and drought effects are significant when CIs do not overlap with zero. The values next to effect sizes stand for the number of studies and the number of unique cases, respectively. (Online version in colour.)

**Figure 2.**
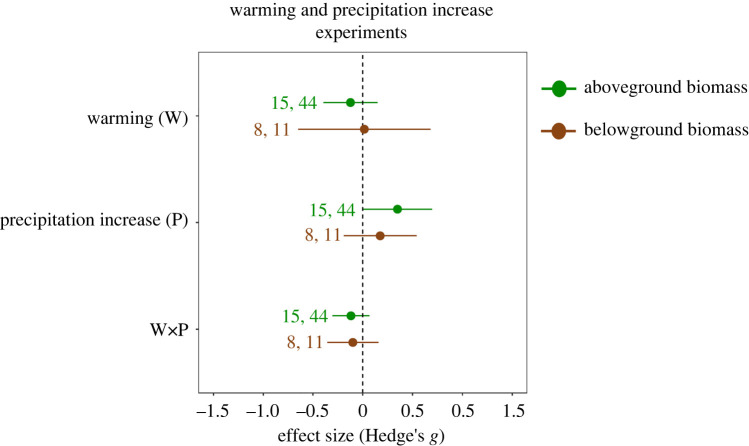
Mean effect sizes ± 95% CIs for experimental warming and increased precipitation on aboveground (upper effect sizes) and belowground (lower effect sizes) plant biomass. Warming and increased precipitation effects are significant when CIs do not overlap with zero. The values next to effect sizes stand for the number of studies and the number of unique cases, respectively. (Online version in colour.)

Drought reduced aboveground plant biomass (effect size (Hedge's *g*) = −0.89, 95% CI = −1.40, −0.37), but had no effect on belowground plant biomass (CI overlapping with zero, figure 1, details in table 1) despite significantly high heterogeneity among studies (table 1). In studies that combined warming and drought treatments, warming did not significantly affect above- or belowground biomass (CI overlapping with zero, figure 1, table 1). On average, combined effects of warming and drought on above- and belowground biomass did not differ from zero, indicating that individual effects of warming and drought were additive (figure 1, table 1). Increased precipitation slightly enhanced the aboveground biomass (effect size (Hedge's *g*) = 0.34, 95% CI = 0.0002, 0.69; figure 2, details in table 1) along with significantly higher heterogeneity among studies (table 1). Similar to studies examining warming and drought effects, warming did not significantly affect above- or belowground biomass in studies that combined warming and increased precipitation, and the interactive effect indicated that effects of warming and increased precipitation on above- and belowground were additive (CI overlapping with zero, figure 2, table 1).

**Table 1.** Effect size (Hedge's *g*) and standard errors (s.e.) of individual and interactive effects of warming and precipitation extremes (drought and increased precipitation) on aboveground and belowground plant biomass. Heterogenity test statistics *Q*, combined with respective degrees of freedom and *p*-value, are also provided. The effect of study identity as random intercept in our models is listed as their variance. Italicized effect sizes are statistically significant.

	effect size (s.e.)	CIs (95%)	test for heterogeneity (*Q*)	d.f.	*p*-value	variance component (study)
aboveground biomass						
warming (W)	0.103 (0.223)	−0.347, 0.553	176.492	79	<0.001	0.931
drought (D)	*−0.895 (0.263)*	*−1.407, −0.375*	219.174	79	<0.001	1.248
W × D	0.027 (0.110)	−0.188, 0.243	132.762	79	<0.001	0.179
warming (W)	−0.123 (0.138)	−0.395, 0.148	55.545	43	0.095	0.096
precipitation increase (P)	*0.347(0.177)*	*<0.001, 0.695*	61.848	43	0.031	0.258
W × P	−0.119 (0.094)	−0.303, 0.065	65.983	43	0.013	0.042
belowground biomass						
warming (W)	−0.101 (0.240)	−0.571, 0.368	73.692	34	<0.001	0.547
drought (D)	−0.049 (0.094)	−0.233, 0.135	25.593	34	0.849	0.000
W × D	0.087 (0.111)	−0.132, 0.306	39.973	34	0.221	0.081
warming (W)	0.015 (0.338)	−0.647, 0.678	21.037	10	0.020	0.549
precipitation increase (P)	0.174 (0.186)	−0.189, 0.539	7.587	10	0.347	0.009
W × P	−0.100 (0.130)	−0.356, 0.156	6.708	10	0.752	0.000

**Figure 3.**
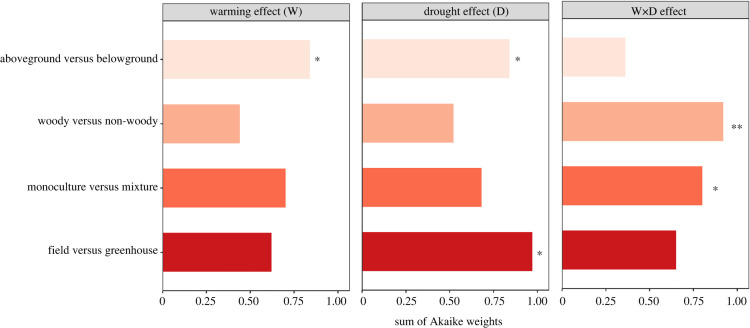
Sum of Akaike weights of four moderator variables from multi-level meta-analytic models for biomass responses in experiments examining warming and drought effects. The higher the Akaike weights, the greater is the importance of the variable in explaining the variation of an effect size. The statistical significance of a given moderator variable is indicated by an asterisk, and when those without any asterisk sign are non-significant. Asterisks represent *p*-values < 0.05 (*) or *p*-values < 0.01 (**). (Online version in colour.)

**Figure 4.**
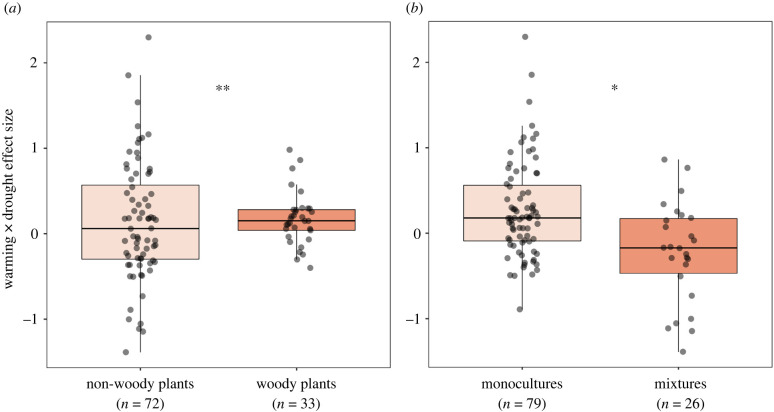
(*a*) Difference in the warming × drought interaction effect size between woody and herbaceous (non-woody) plant responses. (*b*) Difference in the warming × drought interaction effect size between plant monocultures and mixed plant communities. Positive or negative values indicate combined effects that are more positive or negative than expected based on single effects, respectively. Asterisks represent *p*-values < 0.05 (*) or *p*-values < 0.01 (**). Boxplots show the median effect size (horizontal line), first and third quartiles (rectangle), 1.5 × interquartile range (whiskers) and all effect sizes (as black dots). (Online version in colour.)

**Figure 5.**
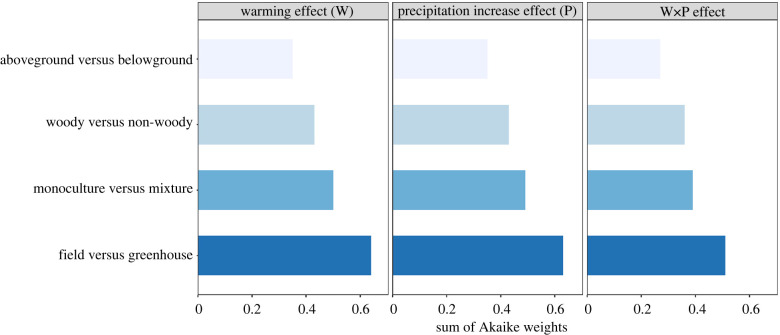
Sum of Akaike weights of four moderator variables from multi-level meta-analytic models for biomass responses in experiments examining warming and increased precipitation effects. The higher the Akaike weights, the greater is the importance of the variable in explaining the variation of an effect size. (Online version in colour.)

In studies combining warming and drought, warming effects depended most strongly on whether biomass responses were measured above- or belowground (figure 3), with more positive warming effects on aboveground than on belowground biomass, although differences between responses of aboveground and belowground biomass were small (electronic supplementary material, figure S3). Variation in plant biomass responses to drought was best explained by experimental type (greenhouse versus field), as impacts of drought on plant biomass were more negative in greenhouse studies than in field studies (figure 3; electronic supplementary material, figure S4). Moreover, drought affected aboveground biomass more negatively than belowground biomass (figure 3; electronic supplementary material, figure S4). The warming × drought interaction effect varied most between woody and herbaceous plants (figures 3 and 4). Warming and drought interactively affected herbaceous plants more negatively than woody plants (figure 4). Moreover, the warming × drought interaction effect size also differed between plant monocultures and mixtures (figures 3 and 4), as the interaction effect size was slightly more negative for plant mixtures than for plant monocultures (figure 4). Across experiments that combined warming and increased precipitation treatments, we found that experiment type (greenhouse versus field) consistently was the most important moderator in explaining the variation in all three effect sizes (figure 5), but these differences in plant responsiveness between greenhouse and field studies were nevertheless not significant (*p*-value > 0.05).

## 4. Discussion

Determining terrestrial plant biomass responses to multiple global change factors is crucial to improve understanding of future plant communities and carbon dynamics in terrestrial ecosystems [6,54–56]. Towards this end, we performed a meta-analysis of experimental studies that examined the interactive effects of warming and precipitation extremes on plant biomass responses above- and belowground. In line with previous meta-analyses, our meta-analysis shows that drought negatively affected aboveground plant biomass, while increased precipitation had a slightly positive effect on aboveground plant biomass [10,13]. However, in contrast, a previous meta-analysis showed that above- and belowground biomass responses to both precipitation extremes are comparable [13], while in our analysis belowground biomass was not significantly affected by either of the precipitation extremes. It must be noted that our dataset included more measurements for above- than for belowground biomass (figure 1), and most of the strongly negative drought effect sizes came from studies that reported aboveground biomass responses (electronic supplementary material, data S2), but not belowground biomass responses. Therefore, the limited effect of drought on belowground biomass may at least in part be explained by a bias towards aboveground biomass measurements in warming and drought experiments. Experimental warming overall did not affect above- or belowground biomass, unlike what was found in previous meta-analyses [10,13]. Moreover, effects of precipitation extremes did not depend on interactions with warming, indicating that warming did not strengthen or weaken effects of increased precipitation or drought on plant biomass [10]. This was true despite the fact that we only included global change studies that tested warming and precipitation extremes interactively, as opposed to previous meta-analyses, which pooled single-effect and combined-effect studies [10,13]. Thus, although it included fewer studies than previous meta-analyses [10,13], our meta-analysis further confirms that warming and precipitation extremes on average exert additive effects on above- and belowground plant biomass.

Given that interactive effects of warming and precipitation extremes are found in individual studies (e.g. [14]), we examined whether specific biotic characteristics may explain variation in plant biomass responses to single or combined impacts. Indeed, our results show that the strength of interaction effects between warming and drought differs between woody and herbaceous plants as well as between plant monocultures and mixed plant communities. These results highlight that biotic contexts, such as plant growth form and plant community type, are important to consider when predicting plant biomass responses to combined effects of warming and drought [57,58].

While we did not find a significant interactive warming × drought effect on plant biomass across studies, we found that the interactive effect of warming and drought was more negative for herbaceous plants than for woody plants, indicating that herbaceous plants on average suffer more from drought under warm conditions than woody plant species do. Interestingly, the experimental systems in which interactive warming × drought effect sizes were most negative (Hedge's *g* lower than −1) were all mixed herbaceous communities that included grasses (electronic supplementary material, data S2). Possibly, warming further worsens drought effects on shallow-rooted, herbaceous plant species [19] and therefore may most negatively affect grass species [59,60], while woody plants may have overcome such adverse effects at least for a short duration owing to their greater ability to tolerate initial water shortages [28]. It should be noted that most studies in our meta-analysis examined drought responses for a limited amount of time, e.g. for a single growing season or shorter. However, under severe and prolonged drought, woody species will likely also show strong negative responses [3,61].

Differences between woody and non-woody species in their biomass responses may also be explained by underlying variation in life-history traits, e.g. those associated with the leaf economics spectrum [62]. We explored this possibility for responses among plant monocultures, by examining the correlations between values for specific leaf area (SLA) (extracted from the TRY database; [63]) and single factor and interaction effect sizes (see electronic supplementary material, figure S6). These analyses suggest that slow-growing plant species (i.e. species with low SLA values) tend to show a more positive response to interactive effects of warming and drought than fast-growing plant species (electronic supplementary material, figure S6), which is in line with the higher resistance against climate extremes associated with conservative traits [39,42]. However, these correlations can only be confirmed through examining a larger number of species. Species with low SLA values also tended to show more positive responses to increased precipitation (electronic supplementary material, figure S6), but the strong variation in responses among species prevents any conclusive interpretation of this relationship. Finally, it must be noted that variation in biomass responses among woody plant species could depend on their life stages, as younger plants, for example, respond more negatively to drought than mature individuals [64]. Moreover, as with increasing plant age differential responses in total biomass may become more difficult to detect, we suggest that examination of both biomass productivity and absolute biomass may yield a more complete understanding of global change impacts across plant systems. Therefore, including more information on drought treatments (i.e. length and severity), the study plant system (life stage and structural traits), and analysing various measures of biomass responses in future experimental and synthesis work may yield further insights into variation in drought responses among woody and herbaceous plants.

Our results also showed that the warming × drought effect size was more positive in plant monocultures than in mixed plant communities, indicating that, under warming, monocultures were less negatively affected by drought than plant mixtures. This is surprising, as plant diversity typically mediates negative drought impacts on biomass production [35,37–39], most importantly owing to the presence of a broader range of water-use strategies in diverse communities [39,42]. Moreover, high-diversity communities have been shown to benefit more from warming than monocultures [36], likely owing to positive plant diversity effects on soil water-holding capacity under warm conditions, as well as more strongly positive biomass responses to the warming-induced extension of the growing season [36]. This also would suggest that diverse communities may be less negatively affected by combined applications of drought and warming than monocultures, as drought effects are likely better mitigated. However, our results showed the opposite effect, although only subtly. Possibly, this effect may be driven by the same strongly negative effects of combined applications of drought and warming on mixed herbaceous communities that contained grasses, which also partly explained the difference in interactive global change effects between woody and herbaceous plant systems. Therefore, future studies should examine how functional community composition of plant communities relates to biomass responses to warming and precipitation extremes. Importantly, owing to the limited number of studies, we only differentiated between studies as monocultures and mixed plant communities, regardless of diversity level. A recent synthesis study indicated that biomass responses to global change such as warming are likely to depend on the number of plant species within a community [65]. Therefore, we do not draw any strong conclusions on how biomass responses to single or combined impacts of warming and drought change with increasing plant diversity. Nevertheless, our results encourage future studies to examine whether the interactive effects of warming and drought on plant biomass become more important along the plant diversity gradient.

Our results further showed that drought effects on plant biomass were significantly more negative in greenhouse studies than in field studies. Average relative reductions of water addition between control and drought-exposed experimental units indicated that treatment severity was stronger in greenhouse (42% reduction) than in field experiments (31% reduction; electronic supplementary material, data S1), although changes in water additions were not reported for all studies, which prevented us from performing any further analysis related to exact drought manipulations. Therefore, we suspect that more negative responses in greenhouse studies may in part be explained by differences in the strength of drought treatments between greenhouse and field experiments. However, the observed differences in plant responses to drought between greenhouse and field studies could also be partly explained by the limited ability of plants to express phenotypic plasticity (e.g. deeper root foraging) to overcome drought stress in (shallow) pot experiments, and by the limited capacity of potted soils to buffer environmental changes [32,33]. Experiment type (greenhouse or field) also consistently explained most of the variation in plant biomass responses to individual or combined applications of warming and increased precipitation, although these differences in effect sizes were not significant. Overall, this suggests that outcomes of studies on changes in water availability are to some extent affected by the type of experiment, indicating the need for carefully considering whether implemented treatments in greenhouse experiments resemble the conditions that plants experience under natural conditions[66,67].

In conclusion, our meta-analysis confirms that combined effects of warming and precipitation extremes on plant performance are overall additive and advance our current understanding of how plants' woodiness and community context could play an important role in explaining the combined effects of warming and drought on plant biomass. We suspect that these results are mostly valid at plant population and community levels as most studies included in our meta-analysis report biomass responses to warming and precipitation extremes at these two ecological scales. The clear additive effects of warming and both types of precipitation extremes on both above- and belowground plant biomass suggest that plants have distinct strategies to overcome these potential abiotic stresses. However, in our meta-analysis, we only examined the importance of a couple of plant biotic and experimental parameters of the examined plant systems that may help explain plant responses to climate change, while, for example, the incorporation of individual- and community-level shoot and root traits may further help to improve our general understanding of plant responses to drought [41]. Finally, our results suggest that precipitation treatments in greenhouse experiments are often stronger than in more realistically set-up field experiments. Therefore, to improve the predictability of plant responses to warming and precipitation extremes, future experiments should be focussed on examining plant responses to climate change across all important plant traits and carefully simulate climate change treatments.

## Data Availability

We provide the database with extracted data from previously published studies (electronic supplementary material, data S1) as well as the effect size data (electronic supplementary material, data S2) as electronic supplementary material [68].
